# The regulatory role and clinical application prospects of circRNA in the occurrence and development of CNS tumors

**DOI:** 10.1111/cns.14500

**Published:** 2023-11-12

**Authors:** Bo Zhang, Hao Zhang, Zeyu Wang, Hui Cao, Nan Zhang, Ziyu Dai, Xisong Liang, Yun Peng, Jie Wen, Xun Zhang, Liyang Zhang, Peng Luo, Jian Zhang, Zaoqu Liu, Quan Cheng, Renjun Peng

**Affiliations:** ^1^ Department of Neurosurgery, Xiangya Hospital Central South University Changsha China; ^2^ National Clinical Research Center for Geriatric Disorders, Xiangya Hospital Central South University Changsha China; ^3^ Department of Neurosurgery, The Second Affiliated Hospital Chongqing Medical University Chongqing China; ^4^ MRC Centre for Regenerative Medicine, Institute for Regeneration and Repair University of Edinburgh Edinburgh UK; ^5^ Department of Psychiatry, The School of Clinical Medicine Hunan University of Chinese Medicine Changsha China; ^6^ College of Life Science and Technology Huazhong University of Science and Technology Wuhan China; ^7^ Teaching and Research Section of Clinical Nursing Xiangya Hospital of Central South University Changsha China; ^8^ Department of Geriatrics, Xiangya Hospital Central South University Changsha China; ^9^ Department of Oncology, Zhujiang Hospital Southern Medical University Guangzhou China; ^10^ Department of Interventional Radiology The First Affiliated Hospital of Zhengzhou University Zhengzhou China

**Keywords:** circRNA, CNS tumors, chemotherapy, immunotherapy, radiotherapy

## Abstract

**Background:**

Central nervous system (CNS) tumors originate from the spinal cord or brain. The study showed that even with aggressive treatment, malignant CNS tumors have high mortality rates. However, CNS tumor risk factors and molecular mechanisms have not been verified. Due to the reasons mentioned above, diagnosis and treatment of CNS tumors in clinical practice are currently fraught with difficulties. Circular RNAs (circRNAs), single‐stranded ncRNAs with covalently closed continuous structures, are essential to CNS tumor development. Growing evidence has proved the numeral critical biological functions of circRNAs for disease progression: sponging to miRNAs, regulating gene transcription and splicing, interacting with proteins, encoding proteins/peptides, and expressing in exosomes.

**Aims:**

This review aims to summarize current progress regarding the molecular mechanism of circRNA in CNS tumors and to explore the possibilities of clinical application based on circRNA in CNS tumors.

**Methods:**

We have summarized studies of circRNA in CNS tumors in Pubmed.

**Results:**

This review summarized their connection with CNS tumors and their functions, biogenesis, and biological properties. Furthermore, we introduced current advances in clinical RNA‐related technologies. Then we discussed the diagnostic and therapeutic potential (especially for immunotherapy, chemotherapy, and radiotherapy) of circRNA in CNS tumors in the context of the recent advanced research and application of RNA in clinics.

**Conclusions:**

CircRNA are increasingly proven to participate in decveloping CNS tumors. An in‐depth study of the causal mechanisms of circRNAs in CNS tomor progression will ultimately advance their implementation in the clinic and developing new strategies for preventing and treating CNS tumors.

## Graphical abstract

The precise risk factors and molecular mechanisms underlying CNS tumors remain unverified. Growing evidence suggests that Circular RNAs (circRNAs) have numerous significant biological functions in disease progression. In this review, we provide an overview of the relationship between circRNAs and CNS tumors and discuss the diagnostic and therapeutic potential of circRNAs in CNS tumors.

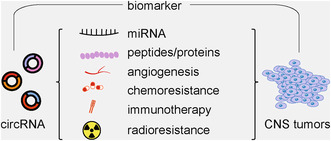

## 1 BACKGROUND

Central nervous system (CNS) tumors are a cluster of tumors that develop from the spinal cord and brain and are classified by the WHO as fourteen types.^1^ CNS tumors worldwide account for disproportionate morbidity and mortality rates.^2^ In 2021, the World Health Organization (WHO) classification scheme incorporated specific molecular changes into diagnosing most tumors. Although several treatment approaches have been developed, CNS tumors remain relatively intractable cancers, with mortality rates unaltered for decades.^3^ To understand how CNS tumors progress and develop, molecular mechanisms of CNS tumors must be elucidated to uncover definitive biomarkers for diagnosis, prognosis, and therapy. Several ncRNAs, consisting of microRNAs (miRNAs) and small interfering RNAs (siRNAs), are reported to participate in the malignant process of CNS tumors. They can be applied to be valuable in clinical therapy, prognosis evaluation, and early prognosis.^4^, ^5^ Recently, circRNA, a large class of ncRNAs derived from back splicing, provided new insights for exploring comprehensive pathogenic mechanisms of CNS tumors. CircRNA molecules were initially detected in the Sendai virus.^6^ Using electron microscopy, Hsu and Coca‐Prados discovered circRNA in eukaryotic cells 3 years later.^7^ In the beginning, the circRNA was considered “junk.” With bioinformatics and high‐throughput RNA‐seq development, researchers have determined many circRNAs in eukaryotes in the last few years. The latter were found to have cell‐specific and tissue‐specific expression patterns.^8^, ^9^ It was proved by more and more evidence that circRNAs were associated with developing multiple diseases, like cardiovascular diseases,^10^ Alzheimer's disease,^11^ and various cancers. Increasing research has verified circRNAs as temporal and spatial‐specific molecules in the CNS and are tissue‐specific and developmentally dependent.^12^, ^13^, ^14^ In CNS tumors, circRNAs could be biomarkers for treatment and diagnostic purposes. This review aims to summarize current progress regarding the molecular mechanism of circRNA and to explore the possibilities of clinical application based on circRNA in CNS tumors.

### 1.1 Biogenesis and modulation of circRNAs

**FIGURE 1:**
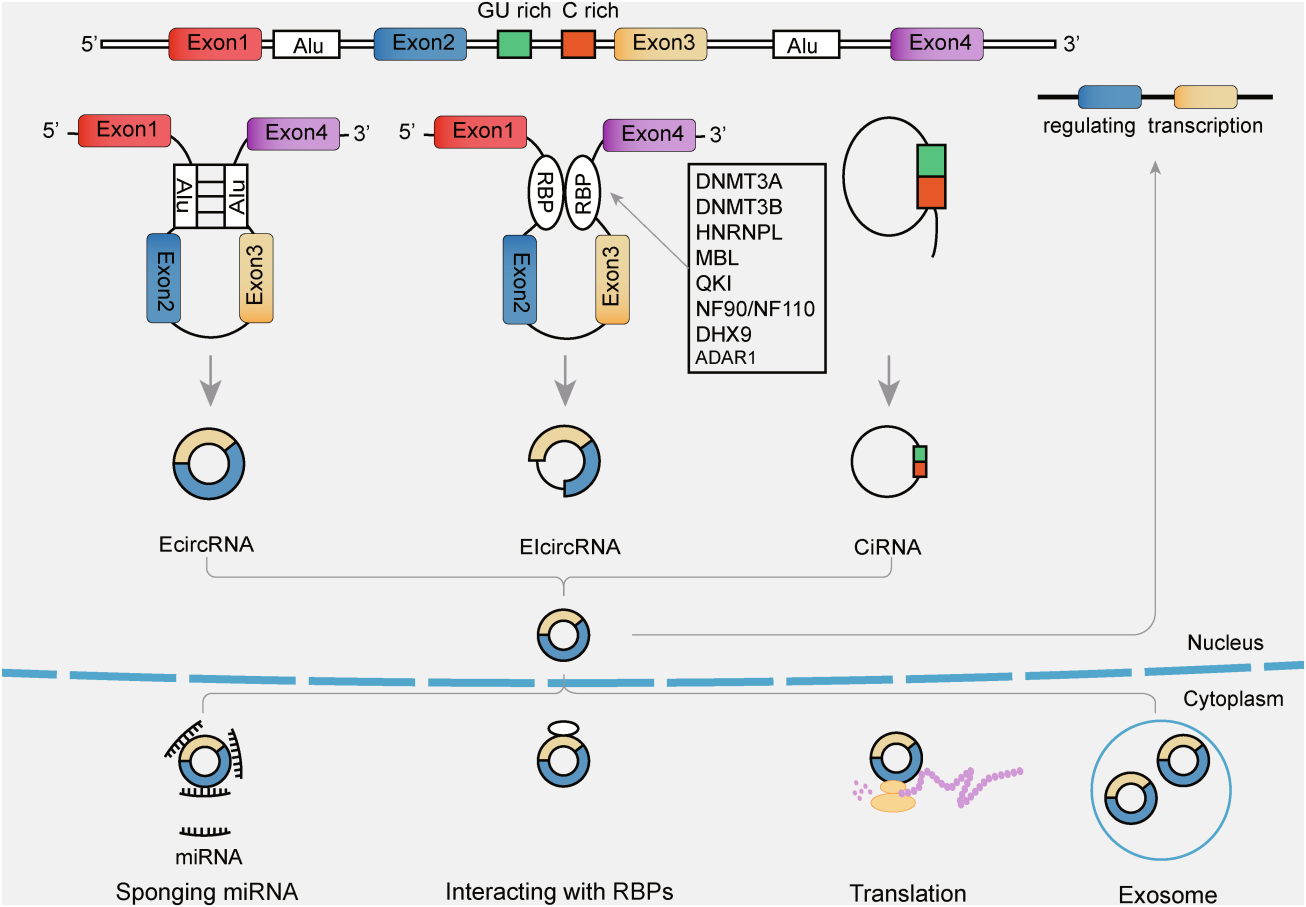
Biogenesis forms, regulation, and function of circRNAs. Three mechanisms of circRNAs biogenesis consist of EcircRNAs, EIcircRNAs, and CiRNAs. Factors regulating the biogenesis of circRNAs, including Alu elements and RBPs. The functions of circRNAs consist of sponging to miRNAs, regulating gene transcription and splicing, interacting with proteins, and encoding proteins/peptides and exosomal circRNAs.

A circRNA is a single‐stranded, covalently closed RNA within the ncRNA group, and the molecule is mainly generated via a back‐splicing process. Different from canonical RNA splicing, in which the intron between neighbor exons was removed by a 5′ site combining the 3′ site cross an intron, some pre‐mRNAs may sustain back‐splicing: splice donors join upstream splice acceptors across multiple exons to form covalently closed circRNAs.^15^ Around four out of five circRNAs are derived from cytoplasmic exons of protein‐coding genes,^14^ as well as circRNA biogenesis depends on the canonical splicing mechanism.^16^ The biogenesis mechanisms of circRNAs can be grouped into three groups (Figure 1): EcircRNAs or exonic circRNAs,^17^, ^18^ EIcircRNAs or exon‐intron circRNAs,^19^ and CiRNAs.^20^

The process of back‐splicing is affected by various factors, such as inverted repeat elements or the binding of RNA‐binding proteins (RBPs). For example, exon cyclization depended on inverted repeated Alu pairs.^17^ In addition, RNA pairing between flanking introns or within individual introns can modulate the former.^17^ According to recent reports, RBPs might regulate the formation of circRNAs such as HNRNPL,^21^ ADAR1,^22^, ^23^ DHX9,^24^ MBL/MBNL1,^25^ QKI,^26^ NF90/NF110,^27^ and DNMT3A/DNMT3B.^28^ This research indicated that epigenetic mechanisms govern the observed circRNA expression changes. It is still unclear how circRNAs are formed and how they are regulated during circularization. These processes need to be investigated in more depth.

Since there are no 3′ or 5′ ends in circRNA sequences, many classical RNA degradation pathways are inaccessible to circRNAs. In recent years, a number of circRNA degradation approaches were discovered. A study proved that RNase L could globally catalyze circRNA degradation.^29^ A research proved that UPF1 and its associated protein G3BP1 regulate circRNAs in a structure‐mediated decay manner.^30^ In addition, Hansen et al. revealed that miR‐671 regulated the cleavage of CDR1as via an Ago2‐slicer‐dependent manner.^31^ The current understanding of circRNA metabolic pathways in vivo is still incomplete, and the detailed mechanisms of circRNA degradation remain to be explored.

### 1.2 Functions of circRNAs

Many researchers identified circRNAs as engaging in several molecular mechanisms of cellular normal or pathophysiology processes. CircRNAs play the role of miRNA sponges in the cytoplasm, one of their most widely researched functions. Upon inhibition of target miRNAs by circRNAs, the modulation of target genes was activated by miRNA response elements.^32^, ^33^ As a result of miRNA‐connected downregulation of mRNA and related molecular mechanisms, circRNAs could be potential in the treatment and progression of malignant tumors. CircACTN4 suppresses miR‐424‐5p to regulate the YBX1/FZD7 axis to facilitate the progression of intrahepatic cholangiocarcinoma.^34^ CircRNAs could compete for splice sites with their parental genes, which would regulate linear splicing.^25^ For example, circSEP3 could bind to its cognate DNA locus and form an R‐loop, leading to transcriptional pausing of linear SEP3.^35^ Not all circRNAs could regulate the parental locus. For instance, transcribed from two different promoters, the expressions of PVT1 and circPVT1 seemed to be controlled independently.^36^ CircRNAs are thought to regulate transcription through several pathways. CircRNAs modulate the transcription of RNA polymerase II through various mechanisms. With their producing locus, circRNAs can form R‐loops that affect transcription and may mediate the activation of transcription factors. A three‐stranded structure containing a DNA: RNA hybrid known as an R‐loop and a single‐stranded DNA can influence DNA transcription, repair, and replication.^37^ Despite Pokemon acting as a tumor suppressor,^38^ circPOK in the nucleus and cytosol promoted the development of tumors through the interaction with ILF2 and ILF3 and the coactivation of ILF2/3, which bound *II6* promoter regions.^39^ Several studies have shown that circRNAs interacted with proteins. It has been proposed that circRNAs can form circRNA‐protein complexes that control cellular activity. Some evidence has shown that several circRNAs interacted with specific RBPs, and some circRNAs were also associated with particular RBPs. CircRNA steatohepatitis‐associated circRNA ATP5B regulator (SCAR) is an example. CircRNA SCAR inhibited the interaction of CypD and mitochondrial ROS (mROS) by binding directly to ATP5B of ATP synthase in the mPTP complex, regulating mROS levels during excessive mPTP opening that was promoted.^40^ Further research on the exact mechanism between RBPs and circRNA is continuing. CircRNAs, which are stable, are identified as widely expressed in exosomes. For example, exosomal circRNA 0001445 was reported to be highly expressed in glioma.^41^ Exosomes transport molecules across the cell membrane and play roles in tumor progression, metabolic regulation, immune response, and treatment resistance.^5^ Furthermore, exosomes can also be used in diagnosing or treating CNS tumors because of their steadiness and particular differential expression patterns. Exosomes seem to show incredible clinical application value, but the molecular mechanism of exosomal circular RNA in CNS tumors is still a young field of research.

CircRNAs were regarded as ncRNAs due to insufficient evidence of encoding proteins.^42^ However, open reading frames (ORFs) binding to translating ribosomes have been found in some circRNAs. The primary translation mode of eukaryotic mRNAs is always translated through the well‐known canonical cap‐dependent translation with the initiation of a 7‐methylguanosine cap joining the 5′ sites of mRNA.^43^ There is increasing evidence that the highly conserved ORFs in circRNAs encode functional peptides independently of the 5′ cap structure, such as by inducing the internal ribosome entry site (IRES),^44^ rolling cycle amplification,^45^ encouraging adenosine methylation (N6‐methyladenosine; m6A)^46^ and more.^47^, ^48^ Transcribed by RNA polymerase II, circRNAs and linear RNAs have the same transcriptional efficiency.^17^ With a unique covalently closed structure, the ORFs of circRNAs could circulate across the splicing site and even beyond its length.^49^ The circRNAs‐encoded peptides/proteins, and their parental genes translated into products have partially identical amino acid sequences. Thus, the former may modulate posttranslational modification of the full‐length protein via competitive binding to enzymes.^50^ For example, MAPK1‐109aa encoded by circMAPK1 could inhibit the activation of MAPK1 via competitively binding to MEK1 and suppressing the phosphorylation of MAPK1.^51^ By identifying the coding potential of circRNAs via bioinformatics methods, we can better understand the behavior of circRNA‐derived proteins. The bioinformatic tools for predicting the coding capacity of circRNAs are collected in Table 1.^52^, ^53^, ^54^, ^55^, ^56^, ^57^, ^58^, ^59^, ^60^, ^61^ Researchers found peptides/proteins encoded by circRNAs connected with CNS tumorigenesis, specifically glioma. Numerous studies have revealed that circRNAs are translatable and are essential in the physiological processes pathological processes of multiple diseases, including myogenesis and multiple cancers.^62^, ^63^, ^64^ As for circRNA‐encoding protein/peptides in the CNS, there are also many related types of research. Amyloid beta(Aβ) peptides are essential in the clinical manifestation and pathogenesis of familial Alzheimer's disease.^65^ Mo et al. showed that circAβ‐a was accurately interpreted as a novel Aβ‐containing Aβ175 polypeptide, which appeared as processed Aβ peptides.^66^ A protein termed p113 encoded by circRNA of the CUT‐like homeobox 1 had been recognized in neuroblastoma cells. Also, p113 is connected to bromodomain protein 4 and Zuotin‐related factor 1 to regulate tumor progression.^67^ These proteins/peptides and their parental genes provide a new target in diagnosing and treating CNS tumors.

**TABLE 1:** Tools for identifying potential coding circRNA.

Name	Annotation	Website	PMID
CircCode	A tool for investigating the translation potential of circRNAs	github.com/PSSUN/CircCode	31,649,739
CircPro	A tool for detecting circRNAs with protein‐coding potential	bis.zju.edu.cn/CircPro	29,028,266
IRESpy	A tool for predicting the internal entry sites of ribosomes using XGBoost	irespy.shinyapps.io/IRESpy/	31,362,694
IRESite	A tool designed to examine the sites where viruses and cells enter their internal ribosomes	www.iresite.org	19,917,642
MiPepid	A machine learning tool for micropeptide identification	github.com/MindAI/MiPepid	31,703,551
CPAT	A tool for alignment‐free coding potential assessment by using a logistic regression model	lilab.research.bcm.edu/cpat/index.php	23,335,781
CircRNADb	A tool with annotations of protein‐coding RNAs found in the human circular RNA database	reprod.njmu.edu.cn/circrnadb	27,725,737
circbank	A tool with standardized nomenclature	www.circbank.cn	31,023,147
TransCirc	A database of circular RNAs that are translatable based on evidence from multiple omics	www.biosino.org/transcirc/	33,074,314
ncEP	An experimentally validated database of ncRNA‐encoded proteins or peptides curated by hand	www.jianglab.cn/ncEP/	32,105,730
ORF Finder	A tool to discover possible ORFs in a sequence	www.cbi.swmed.edu/tech.html	11,814,675

## 2 CIRCRNA AND GLIOMA

Conserved circRNAs are aberrantly expressed in many cancer tissues, which express spatially specific circRNAs and are enriched in the CNS.^22^, ^68^ Circular RNAs are encoded by approximately 30% of transcribed genes in the human brain.^69^ Numerous studies suggest that circRNAs are involved in disease processes, offering a potential therapeutic approach for CNS tumors. Research on circRNAs in CNS tumors has increased recently, although relevant studies have focused on gliomas.

Gliomas are the most common primary intracranial tumors, appearing anywhere within the CNS but predominantly in the brain and arising in the glial tissue.^70^ CircRNAs are shown to be widely involved in the biogenesis and progression of gliomas. Based on the bioinformatics analysis and experiments, thousands of circRNAs were identified that were aberrantly expressed in gliomas compared with normal tissues,^71^, ^72^, ^73^, ^74^ suggesting that differential expression of the molecules may be clinically relevant. One circRNA might function by sponging several miRNAs in glioma to regulate tumor progression. For example, circHIPK3 could sponge miR‐421, miR‐124, miR‐654, miR‐124‐3p, and miR‐524‐5p.^75^, ^76^, ^77^, ^78^, ^79^, ^80^ circPTN sponging miR‐145‐5p, miR‐330‐5p, and miR‐432‐5p promoted glioma growth and regulated Nestin, CD133, SOX9, SOX2, RAB10.^74^, ^81^

**FIGURE 2:**
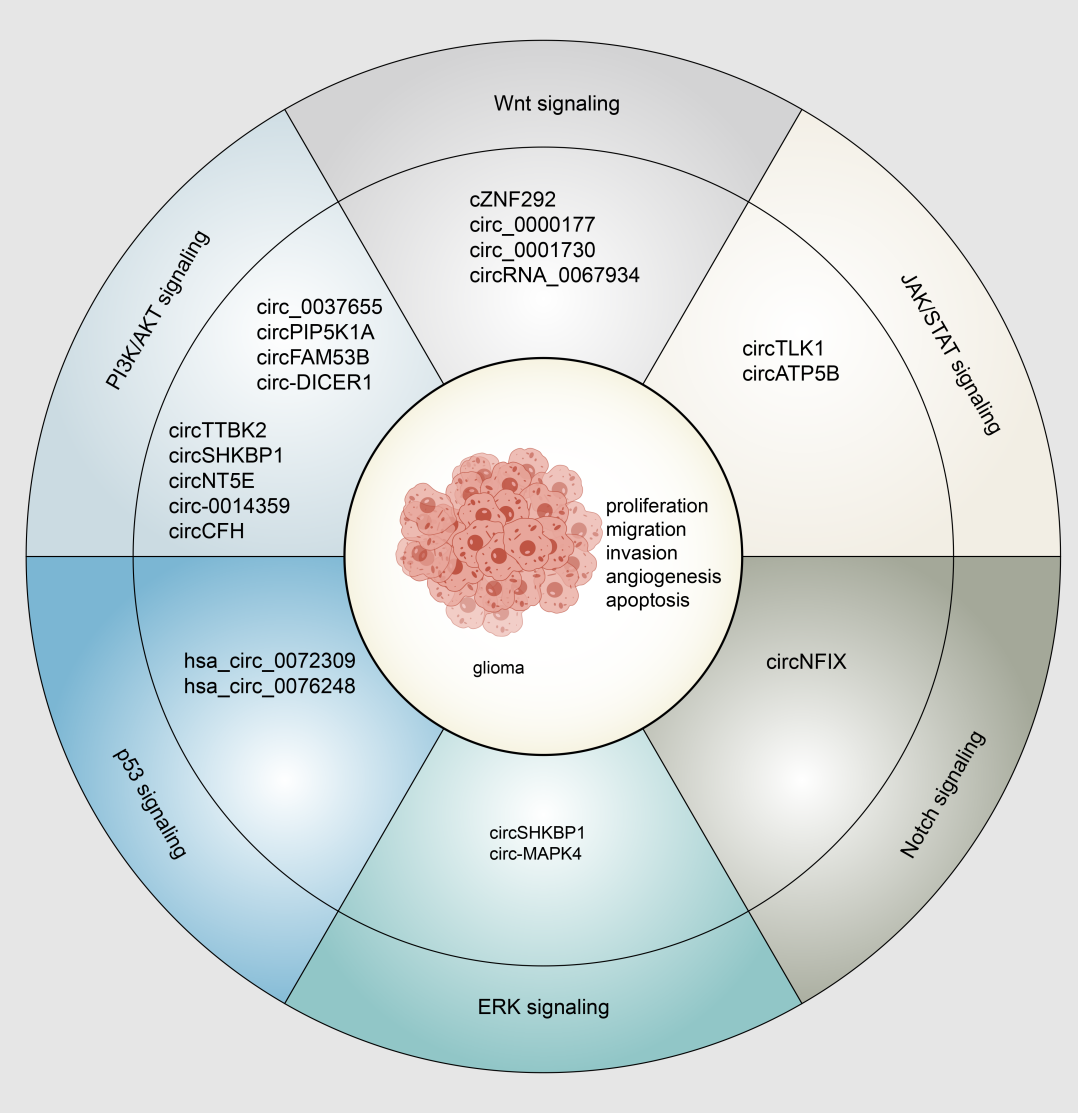
CircRNA and signaling in glioma. Examples of circRNAs regulating signaling to modulate tumorigenesis in gliomas.

A large number of signaling pathways were implicated in glioma tumorigenesis. By regulating sponging miRNAs or target molecules binding to cancer‐relevant signaling pathways, circRNAs are abnormally expressed, widely modulating metastasis, proliferation, and invasion of gliomas (Figure 2).

Various pathological processes are mediated by Wnt signal pathways, including embryonic development, malignant tumors, and tumorigenesis.^82^ Circ_0001730, cZNF292, circRNA_0067934, and circ_0000177 could modulate the miRNA/Wnt/β‐catenin axis and control glioma cell progression.^83^, ^84^, ^85^, ^86^

Multiple cancer types are affected by the PI3K/AKT and ERK signaling pathways.^87^, ^88^ Circ‐DICER1 was reported that it could interact with miR‐103a‐3p/miR‐382‐5p to inhibit ZIC4, which accelerated Hsp90beta in glioma‐exposed endothelial cells (GECs), and Hsp90 promoted the tumorigenesis of GECs binding to PI3K/AKT signaling pathway.^89^ circNT5E, circ‐0014359, circCFH, circ_0037655, circPIP5K1A, and circFAM53B accelerated tumorigenesis via PI3K/AKT signaling pathway by sponging miR422a,^90^ miR‐153,^91^ miR‐149,^92^ miR‐214,^93^ miR‐515‐5p,^94^ and miR‐532‐3p,^95^ implying that these circRNAs may be a regulatory target for the treatment of gliomas.

Among the many intracellular signaling pathways are the JAK/STAT signaling systems, which regulate apoptosis, hematopoiesis, adipogenesis, inflammation, immune fitness, and tissue repair.^96^, ^97^ CircTLK1 was upregulated in glioma cells, sponging miR‐452‐5p/SSR1, thus facilitating JAK/STAT signaling to stimulate glioma malignancy.^98^ Zhao et al. reported that circATP5B modulated the function of HOXB5/IL6 via sponging miR‐185‐5p, JAK2/STAT3 signaling then promoted the proliferation of GSCs.^99^

The Notch signaling pathway can act as an inhibitor and oncogene in various tumors with tissue specificity.^100^ CircRNA modulates the Notch signaling pathway, thus regulating gliomas. According to Xu et al., miR‐34a‐5p interacted with circNFIX and stimulated tumor formation by upregulating NOTCH1 via the Notch signaling pathway.^101^

The p53 is a critical protein significant for inducing cell cycle, apoptosis, and genomic stability arrest, as well as DNA damage, repair, and cellular stress.^102^, ^103^ Hsa_circ_0072309 enhanced the stability of the wild type p53 protein by acting as sponge for miR‐100, thereby impressing p53 ubiquitination. Moreover, in p53 wild‐type GBM, the effect of hsa_circ_0072309 can be counteracted by a P53 or autophagy inhibitor.^104^

It would be highly beneficial if circRNAs were identified as critical players in the molecular mechanism of glioma so that targeted treatments can be developed.

### 2.1 CircRNAs and biomarkers in glioma

In the past, the CNS tumors grading relied solely on histological characteristics. However, advancements in medical science have revealed that molecular markers hold significant potential in offering valuable prognostic insights.^1^ According to the latest WHO guideline, cases of IDH wild‐type diffuse astrocytomas with TERT promoter mutation should be categorized as GBM (Glioblastoma) or CNS WHO grade 4, even if their histological appearance suggests a lower grade.^105^ CircRNAs are highly, conserved expressed in human CNS indicating that the molecules may become crucial biomarkers in numerous diseases, especially tumors.^22^ In the field of gliomas, reports revealed the potential of circRNAs in diagnosis and prognosis. In pinpointing circRNAs and available research, circRNAs function as oncogenes or inhibitors of tumors has significant clinical applications. However, most research has been focused on identifying predictive biomarkers (Table 2). Circ_0034642,^106^, ^107^ circBRAF,^106^ hsa_circ_0008225,^108^ circCHAF1A,^109^ circ_0080229,^110^ circ_0074362,^111^ circ_0001649,^112^ circHIPK3,^76^ and circCPA4^113^ connected to prognosis severity and might be a prognostic biomarker in glioma. NcRNA‐encoded peptides and proteins may provide prognostic information for cancer patients.^114^ CircRNA SHPRH‐encoded SHPRH‐146aa and circRNA FBXW7‐encoded FBXW7‐185aa are negatively associated with poor patient survival.^115^, ^116^

**TABLE 2:** CircRNAs and biomarkers for the diagnosis and prognosis of CNS tumors.

circRNA	Tumor	Expression	Target/mechanism	Downstream pathway	Function	References
circFBXW7	Glioma	Down	Encode FBXW7‐185aa	Reduced the half‐life of c‐Myc by antagonizing USP28‐induced c‐Myc stabilization	Proliferation	28,903,484
					Cell cycle	
circSHPRH	Glioma	Down	Encode SHPRH‐146aa	Protect SHPRH which ubiquitinates PCNA	Proliferation	29,343,848
					Tumorigenicity	
hsa_circ_0001649	Glioma	Down	/	Bcl2/caspase 3	Apoptosis	30,016,668
has_circ_0074362	Glioma	Up	miR‐1236‐3P	HOXB7	Proliferation	30,388,035
					Migration	
					Invasion	
circ_0034642	Glioma	Up	miR‐1205	BATF3	Proliferation	30,551,880
					Invasion	
					Migration	
circHIPK3	Glioma	Up	miR‐124‐3p	STAT3	Proliferation	30,576,808
					Invasion	
circPTN	Glioma	Up	miR‐145‐5p/miR‐330‐5p	SOX9/ITGA5, Nestin, CD133, SOX9, and SOX2	Proliferation	31,511,040
hsa_circ_0008225	Glioma	Down	miR‐890	ZMYND11	Proliferation	32,192,854
					Migration	
					Invasion	
CircRNA PIP5K1A	Glioma	Up	miR‐515‐5p	TCF12,PI3K/AKT signaling pathway	Proliferation	33,413,401
					Invasion	
					Apoptosis	
circATP5B	Glioma	Up	miR‐185‐5p	HOXB5	Proliferation	33,858,489
				SRSF1		
				JAK2/STAT3 signaling		
circCHAF1A	Glioma	Up	miR‐211‐5p	FMR1, HOXC8	Proliferation	34,017,077
					Tumorigenesis	
circ_0080229	Glioma	Up	miR‐1827	MDM2	Tumorigenesis	34,268,375
					Invasion	
circSMARCA5	Ependymoma	Down	/	/	/	33,247,464
circ‐FBXW7	Ependymoma	Down	/	/	/	33,247,464
circRMST	Ependymoma	Up	/	/	/	33,247,464
circLRBA	Ependymoma	Up	/	/	/	33,247,464
circWDR78	Ependymoma	Up	/	/	/	33,247,464
circDRC1	Ependymoma	Up	/	/	/	33,247,464
circBBS9	Ependymoma	Up	/	/	/	33,247,464

### 2.2 Therapeutic potential of circRNAs in glioma

**FIGURE 3:**
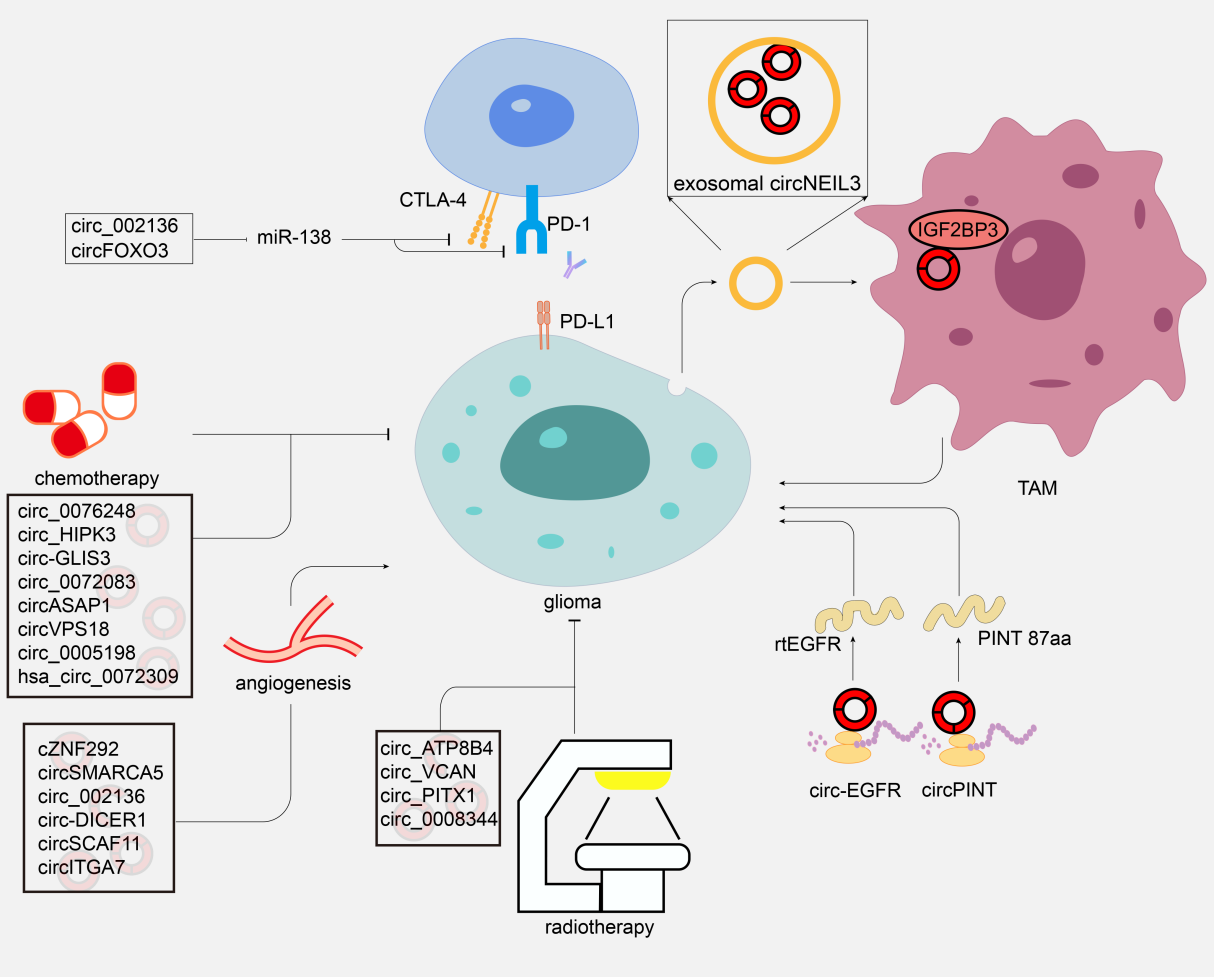
CircRNAs with therapeutic potential in glioma. Circ_002136/circFOXO3 may sponge to miR‐138 and miR‐34a to regulate the immune response of gliomas. Exosomal circNEIL3 could induce IGF2BP3 in TAMs to facilitate glioma progression. rtEGFR and PINT‐87aa could modulate the upregulation of GBM. CircRNAs associated with the sensitivity of chemotherapy and radiotherapy were listed.

Currently, the established approach for treating gliomas involves maximal surgical removal followed by simultaneous radiotherapy and chemotherapy using TMZ, all conducted within a month following the surgical procedure. In addition, bevacizumab and tumor‐treating fields have been applied for glioma treatment. These therapeutic approaches could prolong the median survival of GBM patients but more treatment resource is limited. Finding more glioma treatments is essential. In several studies, circRNAs were regarded as promoters or suppressors to mediate glioma development (Figure 3, Table 3). By functioning as a ceRNA for miR‐433, CircMMP1 silencing suppressed glioma progression in vivo.^117^ Downregulation of circNFIX sponging miR‐34a‐5p inhibited cell propagation and migration.^101^ In vitro and in vivo, circNFIX inhibited glioma progression by increasing miR‐378e and decreasing Ribophorin‐II.^118^ Meanwhile, via sponging miR‐761, hsa_circ_0007534 promoted ZIC5 expression to serve as an oncogene in gliomas.^119^ PTBP1/circRNA_001160/miR‐195‐5p/ETV1 axis is crucial for regulating BTB permeability.^120^ MiR‐7 was identified as a cancer suppressor that precisely decreased tumor‐associated signaling pathways.^121^ circ‐0001801, hsa_circ_0043278, hsa_circ_0006168, circ_0001588, circMELK, circSERPINE2, circLGMN, and circ‐PITX1 were significantly up‐regulated in glioblastoma (GBM) and accelerated the glioma tumorigenesis via miR‐628‐5p/HMGB3,^122^ miRNA‐638/HOXA9,^123^ miR‐628‐5p/IGF1R,^124^ miR‐211‐5p/YY1, and miR‐1281/ERBB4,^125^, ^126^ miR‐593/EphB2,^127^ miR‐361‐3p/miR‐324‐5p/BCL2,^128^ miR‐127‐3p/LGMN,^129^ miR‐379‐5p/MAP3K2^130^ axis. Multiple circRNA were identified that have potential as a therapeutic target in glioma, such as circPITX1,^131^ circ_101064,^132^ circ‐ATXN1,^133^ circPARP4,^134^ circHECTD1,^135^ hsa_circ_0091581,^136^ circZNF609,^137^, ^138^ circ_0001367,^139^, ^140^ circ_CLIP2,^141^ circRNA‐0002109,^142^ circPOSTN,^143^ circCDK14,^144^ and circBFAR.^145^ Modulating the expression of these circRNAs may inhibit glioma development.

**TABLE 3:** Therapeutic potential of circRNAs in CNS tumors.

circRNA	Tumor	Expression	Target/mechanism	Downstream pathway	Function	Clinical significance	References
cZNF292	Glioma	Up	/	Wnt/β‐catenin signaling and related genes including cyclinA, p‐CDK2,VEGFR, EGFR	Proliferation	Therapeutic target	27,613,831
					Cell cycle		
					Angiogenesis		
hsa_circ_0007534	Glioma	Up	miR‐761	ZIC5	Proliferation	Therapeutic target	29,605,301
					migration		
circHIPK3	Glioma	Up	miR‐654	IGF2BP3, CCK8	Proliferation and invasion	Therapeutic target	30,057,315
circNFIX	Glioma	Up	miR‐34a‐5p	Notch signaling pathway	Proliferation	Prognostic biomarker/therapeutic target	30,072,869
circU2AF1	Glioma	Up	miR‐7‐5P	NOVA2	Proliferation	Therapeutic target	30,341,906
					Migration		
					Invasion		
					Apoptosis		
circPINTexon2	Glioma	Down	Encode PINT 87aa	Work as an anchor of PAF1 complex and inhibit downstream genes CPEB1, SOX2, c‐myc	Tumorigenesis	Therapeutic target	30,367,041
hsa_circ_0076248	Glioma	Up	miR‐181a	p53, SIRT1	Tumorigenesis	TMZ sensitivity	30,506,951
					Apoptosis		
					Invasion		
					TMZ sensitivity		
circ‐DICER1	Glioma	Up	miR‐103a‐3p/miR‐382‐5p	ZIC4/HSP90, PI3k/AKT signaling pathway	Proliferation	Therapeutic target	30,621,721
					Migration		
					Angiogenesis		
circATP8B4	Glioma	Up	miR‑766	/	/	Radioresistance	30,664,179
circ_002136	Glioma	Up	MiR‐138‐5p	SOX13/SPON2	Migration	Therapeutic target	30,736,838
					Invasion		
					Angiogenesis		
circ‐PITX1	Glioma	Up	miR‐518a‐5p	IL17RD	Proliferation	Therapeutic target	31,069,865
					Migration		
					Invasion		
circSCAF11	Glioma	Up	miR‐421	SP1,VEGFA	Proliferation	Therapeutic target	31,400,609
					Invasion		
circCPA4	Glioma	Up	let‐7	CPA4	Proliferation, migration	Prognostic biomarker/therapeutic target	31,424,161
circ‐PITX1	Glioma	Up	miR‐379‐5p	MAP3K2	Proliferation	Therapeutic target	31,493,405
					Apoptosis		
circFOXO3	Glioma	Up	miR‐138‐5p/miR‐432‐5p	NFAT5	Migration	Therapeutic target	31,504,797
					Invasion		
circZNF609	Glioma	Down	miR‐134‐5p	BTG‐2	Proliferation	Therapeutic target	31,721,211
					Migration		
circRNA_001160	Glioma	Up	miR‐195‐5p	PTBP1, ETV1	Apoptosis	Therapeutic target	31,862,871
circNFIX	Glioma	Up	miR‐378e	RPN2	Proliferation	Therapeutic target	31,888,753
					Migration		
					Invasion		
					Apoptosis		
circ_101064	Glioma	Up	miR‐154‐5p	PIWIL1	Proliferation	Therapeutic target	31,941,603
					Migration		
					Invasion		
circ‐MAPK4	Glioma	Up	miR‐125a‐3p	p38/MAPK pathway	Proliferation	Therapeutic target	31,992,303
					Apoptosis		
circ_0037655	Glioma	Up	miR‐214	PI3K signaling	Invasion	Therapeutic target	32,001,271
circ_VCAN	Glioma	Up	miR‐1183	/	Proliferation	Radioresistance	32,080,097
					Migration		
					Invasion		
					Apoptosis		
circPITX1	Glioma	Up	miR‐329‐3p	NEK2	Glycolysis	Radioresistance	32,190,004
circ‐ATXN1	Glioma	Up	miR‐526b‐3p	MMP2	Migration	Therapeutic target	32,600,379
circPTN	Glioma	Up	miR‐432‐5p	RAB10	Proliferation	Therapeutic target	32,629,066
					Invasion		
					Glycolysis		
circHIPK3	Glioma	Up	miR‐421	ZIC5	TMZ resistance	TMZ sensitivity	32,644,821
circHIPK3	Glioma	Up	miR‐524‐5p	KIF2A, PI3K/AKT pathway	Proliferation	TMZ sensitivity	32,833,501
					Apoptosis		
					Migration		
					Invasion		
					TMZ resistance		
circMMP1	Glioma	Up	miR‐433	HMGB3	Progression	Therapeutic target	32,918,539
circASAP1	Glioma	Up	miR‐502‐5p	NRAS	TMZ resistance	TMZ sensitivity	32,926,734
circHIPK3	Glioma	Up	miR‐124	CCND2	Proliferation, migration, and invasion	Therapeutic target	33,005,182
hsa_circ_0043278	Glioma	Up	miRNA‐638	HOXA9	Proliferation	Therapeutic target	33,154,193
					Migration		
					Invasion		
circ‐VPS18	Glioma	Up	miR‐370	RUNX1	TMZ sensitivity	TMZ sensitivity	33,188,501
Circ_0005198	Glioma	Up	miR‐198	TRIM14	TMZ resistance	TMZ sensitivity	33,316,781
circ‐EGFR	Glioma	Up	Encoded rtEGFR	Maintained EGFR membrane localization and attenuated EGFR endocytosis and degradation	Tumorigenicity	Therapeutic target	33,325,513
circNFIX	Pituitary adenoma	Up	miR‐34a‐5p	CCNB1	Proliferation	Therapeutic target	33,359,304
					Migration		
					Invasion		
circSKA3	Medulloblastoma	Up	miR‐383‐5p	FOXM1	Proliferation	Prognostic biomarker/therapeutic target	33,408,514
					Migration		
					Invasion		
					Apoptosis		
circPARP4	Glioma	Up	miR‐125a‐5p	FUT4	Proliferation	Therapeutic target	33,520,365
					Migration		
					Invasion		
circ‐UBAP2	Glioma	Up	miR‐1205/miR‐382	GPRC5A	Proliferation	Therapeutic target	33,543,830
					Migration		
					Invasion		
					Angiogenesis		
circHECTD1	Glioma	Up	miR‐296‐3p	SLC10A7	Proliferation	Therapeutic target	33,561,315
					Migration		
					Invasion		
circITGA7	Glioma	Up	miR‐34a‐5p	VEGFA	Proliferation	Therapeutic target	33,962,397
					Invasion		
circ_0072083	Glioma	Up	miR‐1252‐5p	NANOG, ALKBH5	TMZ resistance	TMZ sensitivity	33,975,615
					proliferation		
					Apoptosis		
					Migration		
					Invasion		
hsa_circ_0091581	Glioma	Up	miR‐1243‐5p	RMI1	Proliferation	Therapeutic target	33,976,734
					Migration		
					Invasion		
CircZNF609	Glioma	Down	miR‐1224‐3p	PLK1	Proliferation	Therapeutic target	33,976,745
					Migration		
					Invasion		
hsa_circ_0006168	Glioma	Up	miR‐628‐5p	IGF1R	Proliferation	Therapeutic target	34,024,251
					Migration		
					Invasion		
					Apoptosis		
circ_0001367	Glioma	Down	miR‐431	NRXN3	Proliferation	Therapeutic target	34,035,217
					Migration		
					Invasion		
circ_0001588	Glioma	Up	miR‐211‐5p	YY1	Proliferation	Therapeutic target	34,105,224
					Migration		
					Invasion		
circMELK	Glioma	Up	miR‐593	EphB2	Tumorigenesis	Therapeutic target	34,168,916
Circ_CLIP2	Glioma	Up	miR‐195‐5p	HMGB3	Proliferation	Therapeutic target	34,357,490
circ_0008344	Glioma	Up	miR‐433‐3p	RNF2	/	Radioresistance	34,423,784
CircZNF609	Glioma	Down	miR‐378b	SLC2A1	Apoptosis	Therapeutic target	34,520,391
circSERPINE2	Glioma	Up	miR‐324‐5p,miR‐361‐3p	BCL2	Proliferation	Therapeutic target	34,553,034
circLGMN	Glioma	Up	miR‐127‐3p	LGMN	Proliferation	Therapeutic target	34,582,975
					Invasion		
circ‐GLIS3	Glioma	Up	miR‐548m	MED31	Proliferation	TMZ sensitivity	34,762,494
					Migration		
					Invasion		
					TMZ sensitivity		
circ_0001367	Glioma	Down	miR‐545‐3p	LUZP1	Proliferation	Therapeutic target	34,869,035
					Migration		
					Invasion		
circRNA‐0002109	Glioma	Up	miR‐129‐5P	EMP2	Proliferation	Therapeutic target	34,938,603
					Migration		
					Invasion		
circPOSTN	Glioma	Up	miR‐185‐5p	KIF1B	Tumorigenesis	Therapeutic target	34,974,134
circ_0001588	Glioma	Up	miR‐1281	ERBB4	Proliferation	Therapeutic target	34,982,356
					Migration		
					Invasion		
					Apoptosis		
					Tube formation		
circCDK14	Glioma	Up	miR‐3938	PDGFRA	Proliferation	Therapeutic target	35,002,529
					Migration		
					Invasion		
					Ferroptosis		
circNEIL3	Glioma	Up		IGF2BP3	Tumorigenesis	Therapeutic target	35,031,058
circFAM53B	Glioma	Up	miR‐532‐3p	c‐MET/PI3K/AKT	Proliferation	Therapeutic target	35,100,091
					Colony formation		
					Invasion		
					Epithelial‐mesenchymal transition (EMT)		
circBFAR	Glioma	Up	miR‐548b	FoxM1	Proliferation	Therapeutic target	35,202,487
					Invasion		
hsa_circ_0072309	Glioma	Down	/	p53	Autophagy	TMZ sensitivity	35,212,145
					TMZ sensitivity		

Exosomes have also shown potential in the treatment of gliomas. Via a hnRNPA2B1‐mediated approach, circNEIL3 transported by exosomes infiltrated tumor‐associated macrophages(TAMs), regulating immunosuppressive properties by stabilizing IGF2BP3, an oncogenic protein,^146^ sequentially facilitating glioma progression.^147^ Exosomes transported circRNAs may be a promising treatment modality.

Interestingly, circRNA‐encoded proteins/peptides are reported therapeutic potential in recent studies. Rolling translated EGFR (rtEGFR) was encoded by circ‐EGFR through rolling translation. The protein decreased the degeneration and endocytosis of EGFR. Reduced tumorigenicity and enhanced anti‐GBM effects were observed in brain tumor‐initiating cells with rtEGFR downregulation.^148^ Furthermore, a peptide encoded by circPINTexon2 could inhabit GBM progression.^71^ The proteins/peptides encoded by circRNAs seem to be promising therapeutic targets.

#### 2.2.1 CircRNA and angiogenesis in glioma

Angiogenesis is an essential part of glioma development. Overexpression of the vascular endothelial growth factor (VEGFA) is necessary to microvascular proliferation and blood–brain barrier disruption in glioma.^149^ As an IgG humanized monoclonal antibody that targets VEGFA, bevacizumab has been used in treating recurrent GBM since 2009 and might be sufficient for a clinical benefit in GBM.^150^, ^151^ Affecting tumor angiogenesis by regulating related circRNAs is a possibility for therapying glioma.

Knockdown of circSCAF11 regulated the SP1 expression through sponging miR‐421. SP1 is a transcription promoter of VEGFA. Glioma tumorigenesis was upregulated via the circSCAF11/miR‐421/SP1/VEGFA axis.^152^ CircSMARCA5 stimulated the VEGFA isoforms ratio and acted as an anti‐angiogenic molecule.^153^

CircITGA7 regulated miR‐34a‐5p/VEGFA axis to modulate the proliferation and metastasis of glioma.^154^ circ‐RPL15 could stimulate glioma angiogenesis and tumorigenicity via sponging miR‐146b‐3p to regulate VEGFA.^155^ Silencing of cZNF292, circ‐DICER1, and circ_002136 downregulated glioma tube formation through angiogenesis corresponding genes consisting of EGFR, VEGFRs, SOX13, and ZIC4 in human gliomas.^83^, ^89^, ^156^, ^157^

#### 2.2.2 CircRNA and immunotherapy in gliomas

Immunotherapy of glioma has gained considerable interest over the past years. Immunotherapy constitutes a ground‐breaking specific treatment approach for various tumors, including gliomas. GBM‐induced immunosuppression is a significant plight.^158^ However, several clinical trials of checkpoint inhibitors for GBM showed limited efficacy.^159^ PD‐L1 is implicated in multiple aspects of brain function as a significant immune‐modulating checkpoint related to programmed cell death.^160^ PD‐L1 induced immune response of TME in gliomas and has been evaluated as a predictive factor.^161^, ^162^, ^163^ Recent research has reported that circRNAs could act as ceRNAs to modulate PD‐L1 expression, thereby regulating tumor immune escape in many tumors.^164^ For instance, hsa_circ_0000190 promoted tumor immune evasion by boosting sPD‐L1 expression in non‐small‐cell lung cancer.^165^ Researchers have proved that the inhabitation of IDO, CTLA‐4, and PD‐L1 interacted with increased Treg‐associated long‐term survival in gliomas.^166^ The interaction of ncRNAs and tumor immunity is identified in previous research.^167^ Furthermore, circRNAs may regulate antitumor immunity via modulating molecules to induce signalings consisting of EGFR, STAT3, NF‐Κb, EMT, and PI3K/Akt/mTOR.^168^, ^169^, ^170^, ^171^

Various mechanisms of immune escape in glioma include defects in tumor antigen presentation, alterations in tumor death pathways, metabolic changes, recruitment of immunosuppressive cells, and aberrant molecules in the tumor microenvironment (TME).^172^ Interferon signalings drive antitumor immunity induced by immune checkpoint blockade.^173^ A recent study reported that interferon‐mediated immune dysfunction was crucial in cancer resistance and immune checkpoint blockade.^174^ In sarcoma cells, circCsnk1g3 and circAnkib1 could modulate the immune TME by regulating the expression of interferon‐related genes. At the same time, the circRNAs may control RIG‐I‐mediated pathways to inhabit pro‐inflammatory elements.^175^ CircPIP5K1A could regulate interferon‐regulating factor 4 to facilitate colon cancer development.^176^ However, relative studies on circRNA and interferon signaling in CNS tumors are currently lacking. The informative and inspiring studies in sarcoma and colon cancer are expected to provide some guiding significance in CNS tumors.

CircRNAs could drive immunosuppression and therapeutic resistance to immunotherapy in multiple tumors. In non‐small cell lung cancer, circUSP7 sponging to miR‐934/SHP2 axis and may upregulate resistance to anti‐PD1 immunothrapy.^177^ As for gastric cancer, circDLG1 modulate CXCL12 by sponging to miR‐141‐3p to regulte anti‐PD1 therapy resistance and cancer progression.^178^

Within the TME, gliomas activate extremely systemic and local immune suppression, suppressing anti‐tumor immune defenses.^179^ CircRNAs could participate in anti‐glioma immunity via binding to miRNAs. For example, miR‐138 was capable of targeting immune checkpoints consisting of CTLA‐4 and PD‐1 in gliomas and exerted anti‐glioma effects^180^; sponging to miR‐138, circ_002136, and circFOXO3 could regulate tumor progression and may have potential to target immune checkpoints to modulate immune in gliomas.^157^, ^181^ Further research on the molecular mechanisms circRNAs regulate glioma immunity is very appealing.

#### 2.2.3 CircRNA and chemotherapy in gliomas

In patients with gliomas, the median survival has been prolonged following surgery combined with adjuvant chemotherapy with temozolomide(TMZ) plus radiotherapy.^182^, ^183^ However, the clinical effect of whether the former regimen or receiving bevacizumab in addition to TMZ and radiotherapy followed by TMZ is still disappointing,^149^, ^184^ primarily due to the resistance to TMZ treatment or radiotherapy. Further research into the mechanisms of progression and drug resistance of gliomas, as well as novel therapeutic objective, is crucially required for improving the prognosis of gliomas.

CircRNA‐medicated TMZ sensitivity has been widely verified by regulating specific target genes. For instance, Li and Lan reported that the miR‐548 m/MED31 axis had been shown to promote TMZ‐R glioma progression by sponging circ‐GLIS3.^185^ The overexpression of circASAP1 promoted GBM cell proliferation and TMZ resistance in GBM.^186^ GBM cell proliferation and TMZ resistance were stimulated by overexpression of circASAP1. Suppression of circ‐VPS18 was identified that accelerated TMZ sensitivity via miR‐370 interacted with RUNX1.^187^ Circ_0005198 can absorb MiR‐198 to regulate TRIM14. By silencing TRIM14, TMZ resistance was suppressed, and TMZ‐resistant glioma cells were inhibited from progressing.^188^ Hsa_circ_0072309 upregulated autophagy and stimulated TMZ sensitivity, according to Yuan et al.^104^ Through sponging to miR‐181a, the knockdown of hsa_circ_ 0076248 enhanced TMZ chemotherapy sensitivity by suppressing the expression of SIRT1.^189^ The knockdown of circHIPK3 modulated TMZ sensitivity in glioma via PI3K/AKT signaling pathway.^77^ Furthermore, exosomal circ‐HIPK3 regulated TMZ resistance via miR‐421/ZIC5 axis.^78^ Additionally, the TME may confer chemoresistance with exosomal miR‐1238, making it potential to combat TMZ resistance in GBM.^190^ In gliomas, exosomal circ_0072083 increased NANOG‐mediated demethylation and degradation of miR‐1252‐5p, resulting in TMZ resistance.^191^

#### 2.2.4 CircRNA and radiotherapy in gliomas

Radiotherapy is a critical treatment approach for glioma, and the most commonly used radiological treatment in glioma is conventionally fractionated radiation.^192^ Researchers have found that circRNA contributes to radioresistance in glioma. Zhao et al. demonstrated that circATP8B4 markedly increased in radioresistant U251 cells than in U251 cells. Acting as a miR‐766, circATP8B4 promoted cell radioresistance using RNA‐seq and bioinformatics.^193^ circ_VCAN might reduce glioma radiosensitivity by regulating miR‐1183.^194^ According to Guan et al., miR‐329‐3p bound to circPITX1 inhibited glioma cells’ glycolysis and radioresistance by targeting NEK2.^195^ Circ_0008344 knockdown promoted radiosensitivity in gliomas via the miR‐433‐3p/RNF2 axis.^196^ These circRNAs, which could regulate radioresistance in glioma, have the potential to be a target for enhancing the tumor sensitivity to radiotherapy.

## 3 CIRCRNA AND MEDULLOBLASTOMA

Children are more likely to develop medulloblastoma(MB) than any other malignant brain tumor, making up 8%–10% of childhood brain tumors, comprising 63% of childhood intracranial embryonal tumors.^197^ More than one‐third of patients die within 5 years after diagnosis.^198^

By RNA‐Seq and bioinformatic analysis, Lv et al. identified that 33 circRNAs consisting of circ‐SKA3 and circ‐DTL were aberrantly expressed in MB tissues. There were three upregulated circRNAs and 30 downregulated circRNAs; six circRNAs of them were experimentally validated. Moreover, the overexpression of circ‐SKA3 and circ‐DTL in vitro modulated host genes facilitates the progression of MB. They might serve as novel diagnostic biomarkers and therapeutic targets of MB.^199^ In addition, Wang et al. observed that circSKA3 was highly expressed in MB tissues, and suppression of circSKA3 hindered proliferation, migration, and invasion while stimulating apoptosis by modulating FOXM1 via sponging to miR‐383‐5p.^200^ A recently developed bioinformatic method named “circs” included three circRNA detection pipelines with lower false‐positive rates in contrast to the previous approach with single in silico.^201^ This extremely aggressive disease may be improved through the development of circRNA‐based biomarkers.

## 4 CIRCRNA AND PITUITARY ADENOMA

Tumors of the pituitary gland are called pituitary adenomas. The majority of them are benign; approximately 35%^202^ are invasive, and 0.1%–0.2%^203^ are carcinomas. Change et al. reported that CyclinB1 (CCNB1) and CirculNFIX (has‐circ_0005660) were markedly overexpressed in pituitary adenomas miR‐34a‐5p decreased. By suppressing circNFIX or overexpressing miR‐34a‐5p, pituitary adenoma development was inhibited via targeting CCNB1, implying that the regulatory axis could be potential for treatment.^204^ Du et al. verified that hsa_circ_0001368 significantly upregulated growth hormone‐secreting pituitary adenoma.^205^ Hu et al. confirmed that Hsa_circRNA_102597, either alone or in conjunction with the Ki‐67 index, separated noninvasive and invasive NFPAs and predicted tumor progression/recurrence.^206^

## 5 CIRCRNA AND EPENDYMOMA

Ependymoma is a rare type of CNS tumor with a mean age of diagnosis for adults of 45 years of age.^207^ The clinical prognosis of epithelial tumors varies according to clinic characteristics, histopathology, and molecular phenotypes.^208^ Researchers examined samples using next‐generation sequencing and performed NanoString nCounter experiments. The results showed a notable global decrease of circRNAs in ependymoma. Among them, circSMARCA5 and circ‐FBXW7 were significantly suppressed in ependymomas, which were described as antitumor factors in gliomas, while five circRNAs were remarkably upregulated (Table 2).^209^ This study provided some basis for the relationship between circRNA and ependymomas.

## 6 PERSPECTIVE AND CONCLUSIONS

CNS tumors are multifactorial and multistep comprehensive diseases, and the specific pathogeneses are still not fully understood. There is a substandard prognosis for malignant intracranial tumors, and they are often resistant to various therapies. ncRNAs, including miRNAs, siRNAs, piRNAs, lncRNAs, and circRNAs, contribute significantly to tumor initiation and development. Circular RNAs are more expressed in CNS and involve pathological and physiological processes associated with CNS tumors. Currently, most studies have focused on circRNA in gliomas, which suggests that upregulated circRNA may be more clinically significant than downregulated circRNAs, supported by current studies. CircRNAs may represent a potential treatment target for CNS tumors since they are an important biomarker.

RNA‐based therapeutic approaches have grabbed more attention in infectious disease vaccines, oncology, regenerative medicine, and metabolism diseases. Initially, RNA was not considered a promising therapeutic target because of its relatively short half‐life in vivo. With developing various RNA modifying, packaging, and delivery systems, and deepening clinical research of the RNA molecules, much of this skepticism has been overcome. RNA molecules have many properties, such as folding into complex conformations, binding molecules, and forming catalytic centers, making them potentially therapeutic molecules.^210^ NcRNA‐based drugs are widely studied as new therapeutic targets. More trials of ncRNA are underway. In developing nucleic acid drugs and vaccines, circular RNAs dominate over other RNAs. The covalent closed‐loop structure of circRNAs obstructed exonuclease‐induced degradation, showing dominance over mRNA‐based drugs and vaccines with the vulnerability of being degraded. Additionally, the amount of nucleic acids required for the rolling loop translation of circRNAs is lower than that of mRNAs may lead to lower toxicity.

There have been several applications of mRNA‐based therapeutics, including vaccines for infectious diseases, cancer immunotherapy, protein substitution, and gene editing within cells.^211^ The global COVID‐19 pandemic has dramatically accelerated the development of mRNA vaccines. In 2021, Pfizer‐BioNTech COVID‐19 Vaccine and Moderna COVID‐19 Vaccine received Emergency Use Authorizations.^212^, ^213^ Clinical trials have shown that they provide a long‐lasting immune response, confirming that mRNA‐based vaccines have enormous clinical value in the safety and efficacy.^214^

The exploration of integrating circRNA into clinical applications is an ongoing endeavor. In conclusion, there are nine major approaches to target circRNA. (1) Using circRNA as a target for genetic diagnosis stands out as the most extensively investigated clinical application direction. Searching https://www.clinicaltrials.gov for “circular RNA” showed that the current clinical studies on circRNA are still mainly for biomarkers in acute injury, genetic diseases, and cancers. However, all of these studies were observational and did not include any experimental research. Investigating how to utilize circRNA as a therapeutic target holds significant value. The function of circRNA in tumorigenesis and chemosensitivity of CNS tumors has been demonstrated in recent studies. Exosome‐based circRNA delivery. Exosomes, in addition to nanoparticles, can act as RNA delivery mediators. CircRNA‐targeting siRNA or overexpressed vectors can be delivered by exosomes. Extracellular vesicle long RNAs were identified potentially useful for cancer diagnosis of hepatocellular carcinoma.^215^ (2) RNA interference(RNAi)‐based circRNA knockdown: Numerous investigations have demonstrated that circRNA knockdown is possible with siRNA or shRNA‐mediated RNAi. The FDA of the United States approved the first siRNA drug, patisiran, in 2018 for treating hereditary transthyretin amyloidosis.^216^ Multiple siRNA drugs are now supported, and more are undergoing clinical trials. However, no other ncRNA drugs have yet been approved for medical use by the FDA. In addition, antisense oligonucleotides AON can also mediate circRNA knockdown. However, they are rarely utilized in circRNA interference investigations because their length is typically more significant than that of siRNA or shRNA. But it can also work as a molecule that prevents protein interactions with circRNA. However, this method has several drawbacks, including facile nuclease degradation, poor cell targeting specificity, and nonspecific off‐target effects. (3) CircRNA overexpression based on the carrier: Elevated expression of certain circRNAs might have inhibitory effects on tumors and be associated with longer survival periods in cancer patients. There are numerous vectors available right now that can induce circRNA overexpression. CircRNA overexpression is typically accomplished in vivo using an AAV or lentivirus vector. (4) CircRNA production in a lab setting: Full‐length circRNA can also be produced in vitro using a chemical approach or in vitro transcription. Then it can be cyclized using Splint technology in addition to overexpression through a vector. Li et al. produced circRNA via a permuted intron‐exon element to generate longer circRNA.^217^ Specifically, a D2GFP‐coding circRNA (circRNAD2GFP) was synthesized and reported as long‐lasting compared with those encoded by unmodified mRNA and M1Ψ mRNA. The result could prove that intracellular circRNA has better stability than linear RNA.^217^ However, the product yield of this technological system could be better, and there are issues like immunogenicity. (5) Delivery of circRNA using nanoparticles: Nanoparticles can deliver drugs and other substances to the lesion site. Drug and contrast agent distribution by nanoparticles has been accomplished. How to deliver circRNA into CNS is a challenge. Current attempts to deliver lipid nanoparticles(LNPs) directionally to a specific organ have failed. Specific compounds might be produced across the blood–brain barrier using nanoparticle‐based strategies.^218^ Because of the blood–brain barrier, LNP enrichment into the CNS is more complicated. Adjusting the LNP charge may be an effective method.^219^ Using synthesized Poly (β‐amino esters) to assist the delivery of circMDK siRNA, Du et al. successfully inhibited liver tumor progression in four different liver tumor models.^220^ The most developed nanoparticle system comprises LNP. Through endocytosis, LNP can deliver compounds like siRNA into the cell. Researchers developed a circRNA‐LNP delivery approach to produce a cancer RNA vaccine. The vaccine induced a positively innate immune response and potent antigen‐specific T cell response, which was equally effective compared with modified mRNA‐LNP. In addition, the circRNA‐LNP could combine with adoptive cell transfer therapy to promote the persistence of TCR‐T cells.^217^ (6) Conditional knockdown of circRNA: The Cre‐LoxP system serves a particular and crucial role in animal models and is a crucial technical technique for tissue‐specific gene modification. Cre‐LoxP system can achieve tissue/cell‐specific gene knockout or overexpression in animal models. Various tissue/cell‐specific Cre model animals can be used to implement circRNA targeting therapies. (7) CRISPR/Cas9 to knock out circRNA: According to specific research, circRNA synthesis can be severely inhibited by CRISPR/Cas9 gene targeting of flanking intron regions that regulate ring formation without significantly impacting parent genes, allowing the creation of circRNA‐targeted knockout model animals. (8) CRISPR/Cas13 to knock out circRNA: RNA is the target of the CRISPR/Cas13 system particularly. Studies have demonstrated that the Cas13 system, a crucial new technique for a circRNA‐specific knockdown, may achieve circRNA‐specific knockdown with little impact on parent mRNA. (9) Targeting circRNAs‐translated proteins or peptides: Minority circRNAs are capable of translating into proteins or peptides. As a therapeutic method in clinical medicine, these proteins or peptides demonstrate a new purpose in physiology and pathology. Increasing evidence suggests the tissue‐specific and organ‐specific function of circRNAs, implying that some proteins or peptides encoded by circRNAs may also function in CNS and could be therapeutic targets. Multiple circRNAs with complex functions and peptides and proteins encoded by circRNAs deliver many possibilities for CNS tumor treatment reactions. Therapeutic approaches based on ncRNA showed tremendous potential in multiple diseases.

However, circRNA also faces some challenges to be used in clinical trials. Many studies reported that circRNAs act as miRNA sponges focusing on cancer features that are readily to measure, consisting of cancer cell proliferation, migration, apoptosis, and angiogenesis. Although it is possible that circRNA could sponge miRNA if experiments are not carefully designed, in vitro experiments may only sometimes be representative of physiological conditions.^15^ CircRNA‐based drugs face another challenge of making circRNAs cross cell membranes, so there are adequate circRNA molecules capable of exerting pharmacological functions. But more than the related studies are needed. Last but not the last, it can improve pharmacokinetics and metabolic stability by making chemical modifications.^221^, ^222^

In closing, circRNAs are increasingly proven to participate in developing CNS tumors, but research into circRNAs is still nascent. Obtaining an in‐depth study of the causal mechanisms of circRNAs in CNS tumor progression will ultimately advance their implementation in the clinic. As we learn more about how circRNAs interact with CNS tumor development, new strategies for preventing and treating CNS tumors will emerge.

## AUTHOR CONTRIBUTIONS

QC and RP conceived the original idea and supervised the project. BZ and HZ drafted the manuscript and prepared the figures. ZW, HC, NZ, ZD, XL, YP, JW, XZ, LZ, PL, JZ, and ZL made the tables and helped revise the manuscript. All authors read and approved the final manuscript.

## FUNDING INFORMATION

This work was supported by the National Natural Science Foundation of China (NO.82073893, NO.81703622, and No.81901268), Hunan Provincial Natural Science Foundation of China (NO.2022JJ20095), Hunan Provincial Health Committee Foundation of China (NO.202204044869).

## CONFLICT OF INTEREST STATEMENT

There are no competing interests.

## CONSENT TO PUBLISH

All authors approved the final manuscruipt and agreed to be published.

## Data Availability

Data sharing not applicable to this article as no datasets were generated or analysed during the current study.
